# The impact of antioxidants on antioxidant capacity, DNA fragmentation, and chromatin quality in subfertile men: a randomized clinical trial study

**DOI:** 10.1590/1806-9282.20240211

**Published:** 2024-11-11

**Authors:** Mesut Sengul, Neslihan Hekim, Ramazan Asci, Sezgin Gunes

**Affiliations:** 1 Ondokuz Mayıs University, Faculty of Medicine, Department of Urology – Samsun, Turkey.; 2 Ondokuz Mayıs University, Faculty of Medicine, Department of Medical Biology – Samsun, Turkey.; 3 Ondokuz Mayıs University, Graduate Institute, Department of Molecular Medicine – Samsun, Turkey.

**Keywords:** Infertility, Antioxidant, DNA fragmentation, Chromatin

## Abstract

**OBJECTIVE:**

This randomized clinical trial study aims to investigate the effects of antioxidant food supplementation on the total antioxidant capacity of seminal plasma, sperm DNA fragmentation, sperm chromatin quality, and semen parameters.

**METHODS:**

In this study, a total of 48 subfertile men with moderate physical activity were included. Group 1 was recommended to use the antioxidant supplements, while antioxidant food supplements were not given to Group 2. Total antioxidant capacity, sperm DNA fragmentation, sperm chromatin structure, hormone levels, physical activities, and semen parameters were evaluated before and after treatment. Total antioxidant capacity, sperm DNA fragmentation, and sperm chromatin structure were assessed using ELISA, transferase dUTP nick end labeling, and aniline blue staining, respectively.

**RESULTS:**

Sperm DNA fragmentation (p=0.003) and histone/protamine ratio (p<0.001) were significantly decreased in the patients receiving antioxidant treatment. There was no statistical difference in the total antioxidant capacity values of the post-treatment groups.

**CONCLUSION:**

Antioxidant therapy seems to improve sperm DNA fragmentation and histone/protamine ratios in subfertile patients.

**Clinical Trial Registration Number:**

NCT06042738.

## INTRODUCTION

Infertility has become a social and medical problem and has led to severe challenges to global public health^1^. The pathogenesis of male infertility is complex and multifactorial, and it is often challenging to identify suitable treatment options^2^. Factors such as nutrients, antioxidants, physical exercise, obesity, and paternal age can affect reproductive health and thus impact the quality of semen parameters and sperm DNA fragmentation (SDF) induced by reactive oxygen species (ROS)^3^. It has already been documented that the imbalance between oxidative stress and the antioxidant defense system of the male reproductive tract is related to infertility^4^. Common clinically used antioxidants (individual application or in combination) include retinoids (vitamin A), ascorbic acid (vitamin C), tocopherols (vitamin E), N-acetylcysteine, carnitines, coenzyme Q10, lycopene, melatonin, and antioxidant co-factors such as zinc, selenium, and folic acid^5^. Despite several clinical studies investigating the potential benefits of antioxidant applications in spermatogenesis and sperm protection, the results are contradictory and remain a puzzle. Moreover, the results of a recent randomized clinical trial suggest that combination antioxidant treatment did not improve standard sperm parameters or DNA integrity^6^. Additionally, recent studies suggest that continuous moderate-intensity physical activity may positively affect the oxidant/antioxidant markers in seminal plasma and hormones than continuous high-intensity physical^7^.

The study aimed to investigate the effects of physical activity and antioxidant food supplementation on seminal antioxidant capacity, sperm chromatin quality, SDF index, and sperm parameters.

## METHODS

### Patient selection

A total of 48 subfertile men diagnosed with idiopathic oligoasthenoteratozoospermia (OAT) in the Urology Clinics of Ondokuz Mayıs University (OMU) Medical Faculty Hospital, Türkiye, between March 2021 and November 2021 were included in this study. Ethics committee approval (2021/108) was obtained from the Clinical Research Ethics Committee at OMU. The study was registered with the trial registration number NCT06042738. All participants had a failure to achieve a clinical pregnancy after at least 1 year of unprotected intercourse, and their female partners had had standard gynecological evaluation. All patients were diagnosed with OAT after two semen analyses in the last 6 months. Physical examination findings and hormonal and genetic evaluations of all patients were normal, and couples were accepted as idiopathic infertile. Exclusion criteria of the patients were undergoing sterilization procedures such as vasectomy and patients with varicocele, medical treatment or use of drugs affecting the reproductive function or metabolism, taking multivitamins or herbal products, use of anticoagulants, sperm concentration <5 million/mL, untreated hypothyroidism or uncontrolled diabetes mellitus, and chronic medical conditions such as cancer, heart disease, or cirrhosis.

### Antioxidant food supplement

By computer-assisted simple randomization (www.randomizer.org), patients who agreed to participate in the study were divided into Group 1 (n=24) and Group 2 (n=24). A food supplement containing 2,000 mg carnitine (L-carnitine), 2,000 mg fructose, 932 mg acetyl L-carnitine, 225 mg vitamin C, 115 mg citric acid, 50 mg coenzyme Q10, 14 mg zinc, 115 μg selenium, 3,750 μg vitamin B12, and 500 μg folic acid (Alfasigma Health Science, Trento, Italy) was given daily as an antioxidant to the patients in Group 1. We did not suggest the use of any supplements to the patients in Group 2. All subjects (Groups 1 and 2) were recommended to do moderate physical activity 3–4 days a week for 3 months, for at least 45 min (at least 150 min to 600 METs per week). Patients were followed up throughout the study and received no other treatment.

#### Hormone analyses

Hormone analyses (FSH, LH, prolactin, estradiol, and total testosterone) were performed using the ELISA method by drawing peripheral venous blood samples from the patients between 7 am and 11 am after at least 12 h of fasting.

### Body mass index and physical activity assessment

The body mass index (BMI) of the participants was calculated using the formula kg/m^2^. All patients participating in the study were recommended to get moderate physical activity three to four times a week for 3 months, for at least 45 min. Physical activity assessments were made using the International Physical Activity Questionnaire—Short Form, in which total scores included the sum of walking, moderate-intensity activity, duration (minutes), and frequency (days) of vigorous activity^8^.

### Semen collection and preparation

Semen samples were collected from all patients at the 0th and 3rd months after 2–7 days of sexual abstinence. After incubation for 20–30 min at 37°C, liquefied samples were analyzed according to the World Health Organization (WHO) 2010 guidelines (World Health Organization 2010). Semen analysis was performed using the WHO 2010 guideline throughout the study to ensure data uniformity. Sperm concentration, total sperm count, motility, and progressive motility parameters were assessed. Semen samples were then treated to evaluate the total antioxidant capacity (TAC) in the seminal phase, SDF, and sperm DNA chromatin condensation. For the analysis of SDF, sperm concentrations of samples were adjusted to 2.5×10^6^/mL^9^. Semen samples were centrifuged at 2,000 rpm for 8 min to separate seminal plasma from the sperm pellet. Then, seminal plasma was centrifuged at 8,000 rpm for 10 min at 4°C and was stored at −70°C until TAC measurements. A portion of the semen pellet was fixed for SDF analysis. The remaining pallet was used to determine sperm chromatin condensation with aniline blue staining.

### Measurement of seminal total antioxidant capacity

TAC was measured by the colorimetric assay using the antioxidant assay kit (Cayman Chemical, MI, USA) according to the manufacturer's recommendations^10^. All seminal plasma samples were diluted by 1:10 to 1:20 factor before analysis. Briefly, Trolox standards were diluted sequentially with assay buffer, and then 10 μL of each was added to the 96-well plate. Afterward, 10 μL of each sample was added to the sample wells. All samples and standards were placed in duplicate. Chromogen 10 μL metmyoglobin and 150 μL ABTS (2,2'-Azino-di-[3-ethylbenzthiazoline sulfonate]) were added to both samples and standards. To all wells, 40 μLμL of 441 μM hydrogen peroxide was added, and then the plate was incubated for 5 min on a shaker at room temperature. The absorbances of the standards and samples were measured at 750 nm using a microplate spectrophotometer (Multiscan GO, Thermo Scientific, Finland) after incubation.

Calculations of each standard and sample were made to evaluate the assay. A standard Trolox curve was plotted with the mean absorbance of the standards. The TACs of the samples were calculated according to the formula using the linear regression of that standard curve and the average of the absorbance of the samples: Antioxidant(mM)=[(Sample average absorbance)–(y-intercept)/Slope]×Dilution .

### Assessment of sperm chromatin condensation by aniline blue staining

The sperm pallet of each patient was immediately separated from the seminal plasma. Then, a spot of the sperm pellet was washed twice with 1× phosphate-buffered saline (PBS) (Gibco, NY, USA). A certain amount of sperm suspension was then spread on clean slides, and the smears were air-dried for approximately 15 min. Air-dried smears were fixed with 3% glutaraldehyde for 30 min at room temperature. Afterward, all slides were immersed in a 5% aniline blue solution (Sigma, Steinheim, Germany) diluted in 4% glacial acetic acid (Sigma, Steinheim, Germany) for 15 min^11^. After staining with aniline blue, at least 200 sperm were counted in each slide at 1000× magnification under a light microscope. Light blue spermatozoa receiving no or less staining were considered rich in protamine, and spermatozoa wholly or partially stained in dark blue were assessed as rich in histone.

### Terminal deoxynucleotidyl transferase dUTP nick end labeling for direct detection of sperm DNA fragmentation

SDF was analyzed with TUNEL using the commercial In Situ Cell Death Detection Kit (Roche, Mannheim, Germany) according to the instructions of the manufacturer^11^. After removing the seminal plasma, the pellet was washed with 1× PBS. A hydrogen peroxide (H_2_O_2_) (30%) solution was used as the positive control. H_2_O_2_ was dropped on the fresh semen pellet of one of the samples in each run and incubated for 15 min at 37°C. Afterward, all samples were fixed with 4% paraformaldehyde (PFA) (Merck KGaA, Darmstadt, Germany) for 20 min at room temperature. Then, PFA was removed, and the samples were washed twice with 1× PBS by centrifugation at 2,000 rpm for 8 min. Sperm samples were dropped on the phosphate-buffered (PB) sucrose on polylysine-coated slides. The slides were kept in a humid and dark environment at 4°C overnight. The next day, slides were washed twice with 1× PBS for 5 min and kept in freshly prepared 0.1% sodium citrate and 0.1% Triton X-100 permeabilization solution on ice. For the TUNEL reaction mixture, TUNEL label solution was mixed with the enzyme solution, and 50 μLμL of the mixture was dropped onto the washed slides at the end of permeabilization. Negative controls were formed by adding only label solution in each run. Slides were incubated for 60 min at 37°C in a humid and dark incubator. Afterward, the slides were washed three times with 1× PBS, and a mounting medium with DAPI (Fluoroshield, Sigma, MO, USA) was added and covered with a coverslip. Samples were examined and photographed immediately using an excitation wavelength of 461 nm and 519 nm (green) detection under a fluorescent microscope (BX51, Olympus Life and Material Sciences). Photographs were analyzed using the Image J program (LOCI, University of Wisconsin), and an average of 500 randomly selected spermatozoa in at least three separate areas were counted for each patient.

While blue fluorescence stained all nuclei, green fluorescence only labeled the fragmented sperm DNA. The SDF index was calculated by the number of sperm nuclei stained green/total sperm nuclei identified as blue in the same sample × 100.

### Statistical analysis

The Kolmogorov-Smirnov test was used to evaluate the conformity of dependent and independent continuous variables used in the study to the normal distribution. In the comparison of the arithmetic mean of independent groups (with and without antioxidants), the Student's t-test was used for variables with a normal distribution, and the Mann-Whitney U test was performed for variables that did not fit a normal distribution. Comparisons of arithmetic mean in dependent groups (pre-treatment and post-treatment) were analyzed with a paired t-test for normally distributed variables and a Wilcoxon test for variables found to be non-normally distributed. Statistical data analysis was performed using IBM SPSS Statistics Version 22.0 (Armonk, NY, USA). P<0.05 was considered statistically significant.

## RESULTS

The mean age of infertile patients in Group 1 who received antioxidant supplements was 33.5±5.46 years, while the mean age was 32.8±6.8 years in Group 2 (t=0.42, p=0.674). Similarly, no differences were found between the groups in terms of right (Group 1: 23.3±2.9 mL; Group 2: 22.7±2.9 mL; t=0.24, p=0.811) and left (Group 1: 22.3±4.0 mL; Group 2: 21.6±2.8 mL; t=1.01, p=0.313) testicular volumes. BMI, physical activities, and hormone levels of participants at the beginning of the study and 3 months after the study are given in Table 1. The physical activity of both groups in the third month was higher than the zeroth month scores (p<0.001).

**Table 1:** Body mass index, physical activities, and hormone levels.

Parameters	Group 1 (n=24)	Group 2 (n=24)	Test	p
	Mean±SD	Mean±SD		
BMI—Month 0	28.1±3.6	28.3±3.5	0.24^b^	0.811
BMI—Month 3	27.2±3.2	27.4±3.4	0.32^b^	0.713
	t=0.24^b^; p=0.811	t=0.32^b^; p=0.713		
Physical activity—Month 0	435.3±304.2	498.1±353.4	1.07^a^	0.353
Physical activity—Month 3	852.5±434.5	835.1±469.6	1.09^a^	0.284
	t=1.07^a^; **p<0.001**	t=1.09; **p<0.001**		
FSH (mU/mL) (1.5–12.4) Month 0	6.1±3.2	6.3±2.8	0.23^b^	0.818
FSH (mU/mL) (1.5–12.4) Month 3	6.2±3.2	6.8±3.7	0.64^b^	0.524
	t=0.25^c^; p=0.806	t=0.74^c^; p=0.465		
LH (mIU/mL) (1.7–8.6) Month 0	5.6±1.6	5.8±2.2	0.42^b^	0.678
LH (mIU/mL) (1.7–8.6) Month 3	5.8±1.8	6.9±2.6	1.71^b^	0.093
	t=0.65^c^; p=0.522	t=2.04^c^; p=0.053		
Prolactin (ng/mL) (2.0–18.0) Month 0	12.1±5.7	12.2±4.2	0.65^a^	0.516
Prolactin (ng/mL) (2.0–18.0) Month 3	13.9±5.9	11.8±5.4	1.77^a^	0.076
	t=1.03^d^; p=0.313	t=0.39^d^; p=0.703		
Estradiol (pg/mL) (7.6–43.0) Month 0	28.6±7.1	28.3±5.6	0.17^b^	0.865
Estradiol (pg/mL) (7.6–43.0) Month 3	29.7±6.8	30.9±7.6	0.62^b^	0.54
	t=0.62^c^; p=0.542	t=1.25^c^; p=0.225		
Testosterone (ng/mL) (2.8–8.0) Month 0	454±164.8	419.2±122.9	0.86^b^	0.411
Testosterone (ng/mL) (2.8–8.0) Month 3	498.4±154.4	435.5±125.4	1.55^b^	0.128
	t=1.34^c^; p=0.194	t=0.64^c^; p=0.531		

a Mann-Whitney U test;

b Student's t test;

c Paired t test;

d Wilcoxon test. Statistically significant results are indicated in bold.

While there was a significant increase in the sperm concentrations of both groups in the third month (p<0.001), no change was found in other parameters, including BMI, total motility, and progressive motility between the groups or duration. No statistically significant difference was found between the hormone levels measured at the beginning and the third month in Group 1 and Group 2.

At the end of the third month, the TAC increased slightly, but the increase was not statistically significant in Group 1 (p>0.05). In Group 2, the TAC decreased slightly at the end of the third month compared to the beginning, but the difference was not statistically significant (p>0.05). While there was a statistically significant difference between the initial TAC values of the groups (p=0.027), this difference was also not significant in the third month (p=0.972).

**Figure 1:**
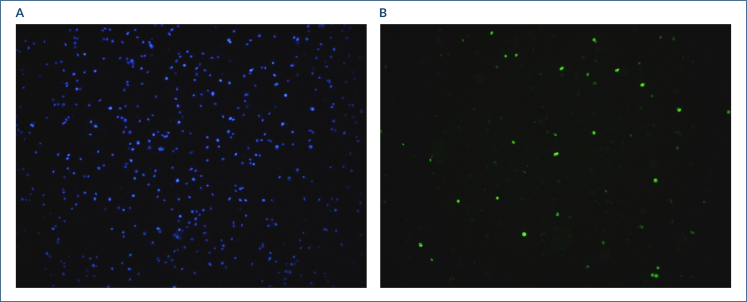
Pictures of transferase dUTP nick end labeling analysis. **(A)** The nuclei of the sperm were stained blue with DAPI. **(B)** The FITC image of the sperm with fragmented DNA in the same area.

A significant decrease in DNA fragmentation index (DFI) was found in Group 1, who received antioxidant food support (p=0.003) (Figure 1). On the contrary, the decrease in DFI was not significant in Group 2 (p=0.078). There was no difference between the DFI's of the groups at month 0 and month 3 (p>0.05) (Table 2).

**Table 2:** Semen parameters, sperm DNA fragmentation, total antioxidant capacity, and chromatin condensation.

Parameters	Group 1 (n=24)	Group 2 (n=24)	Test	p
	Mean±SD	Mean±SD		
Sperm concentration—Month 0	7.2±3.1	7.04±3.1	0.26^a^	0.797
Sperm concentration—Month 3	13.8±6.6	12.2±5.7	0.89^b^	0.378
	**t=5.20** c **; p<0.001**	**t=6.08** c **; p<0.001**		
Total sperm count—Month 0	20.9±12.1	21±14.6	0.55^a^	0.583
Total sperm count—Month 3	22.3±13.1	20.9±12.8	0.48^a^	0.633
	t=0.62^c^; p=0.539	t=0.06^c^; p=0.949		
Progressive motility—Month 0	13.2±9.9	13.1±10.4	0.12^a^	0.908
Progressive motility—Month 3	16.9±9.9	14.3±9.8	1.08^a^	0.28
	t=1.61^c^; p=0.122	t=0.53^c^; p=0.602		
TAC—Month 0	1.6±0.9	2.3±0.9	**2.29** b	**0.027**
TAC—Month 3	2.1±1.2	2±1.3	0.04^b^	0.972
	t=1.15^c^; p=0.263	t=0.68^c^; p=0.501			
SDF, %—Month 0	22.5±11.3	21.3±7.8	0.19^b^	0.853
SDF, %—Month 3	14.7±7.8	17.1±7.4	1.28^b^	0.201
	**t=3.34** c **; p=0.003**	t=1.85^c^; p=0.078			
Histone-rich spermatozoa (%)—Month 0	46.2±14.6	31.3±15.7	**3.61** b	**p<0.001**
Histone-rich spermatozoa (%)—Month 3	26.1±11.8	32.8±15.8	1.44^b^	0.149
	**t=6.59** c **; p<0.001**	t=0.43^c^; p=0.670			

a Mann-Whitney U test;

b Student's t test;

c Wilcoxon test. Statistically significant results are indicated in bold.

**Figure 2:**
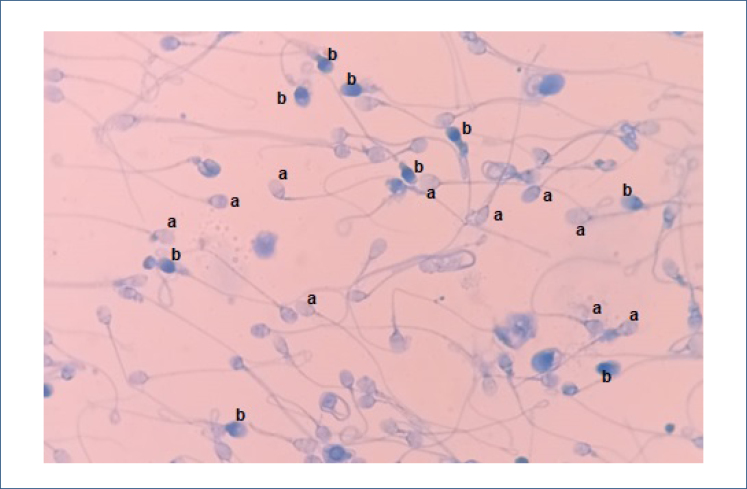
Sperm chromatin condensation analysis using aniline blue staining. (A) Protamine rich spermatozoa. (B) Spermatozoa with histone-rich nuclei.

The percentage of aniline blue-positive spermatozoa (rich in histones) (Figure 2) decreased significantly compared to the baseline in the third month in Group 1, which received antioxidant and nutritional support (p<0.001). On the contrary, there was no significant change in the percentage of protamine-rich spermatozoa in Group 2 (p=0.670). In contrast, the histone-rich spermatozoa were found to be higher in Group 1 compared to Group 2 at the beginning of the study (p<0.001), but there was no difference between both groups at the end of the third month (p=0.149) (Table 2).

## DISCUSSION

In this study, SDF and histone-rich spermatozoa ratios were significantly decreased in the patients receiving antioxidant treatment (Group 1); however, there was also a statistically insignificant decrease in SDF in Group 2 (p=0.078). These findings support the benefit of antioxidant food supplementation in idiopathic male infertility. Treatment of idiopathic male infertility consists of hormonal and non-hormonal empirical medical therapy. The most effective hormonal treatment options are selective estrogen receptor modulators and FSH. Primary non-hormonal treatment agents include carnitines, coenzyme Q10, myoinositol, some vitamins, and trace elements (selenium and zinc)^12^. However, the literature has no consensus regarding the duration of use, dosage, combination, and which subfertile patient groups should be included^13^. Therefore, the study was designed as a non-hormonal treatment randomized and placebo-controlled clinical trial. Antioxidant support was shown to improve primarily sperm motility and also semen parameters^14^. Despite the presence of studies showing a positive correlation between antioxidant treatment on semen parameters in the literature^15–17^, there are also limited studies in which no significant improvement has been demonstrated^18^. Significant improvements in TAC have been reported in most studies conducted in the last ten years on antioxidant support therapy for managing male infertility^19^. Indeed, Alahmar et al. found improvement in seminal parameters and an increase in TAC levels at the end of 3 months performed on 65 oligoasthenozoospermia patients by administering Co-Q10 200 mg/day^15^. Similarly, it has been shown that antioxidant food supplementation given at different times and in different combinations increases sperm count, mobility, and TAC and significantly decreases ROS levels in infertile men^20^. In a separate study examining sperm parameters by administering N-acetylcysteine, it was shown that sperm morphology improved and motility increased, malondialdehyde and DFI levels were decreased in asthenozoospermic men, and TAC levels increased by eliminating protamine deficiency^21^. Conversely, few studies have been published showing no change in TAC levels despite the antioxidant therapy. This study also showed that the TAC level increased statistically insignificant in the group receiving supportive treatment compared to patients who did not receive food supplements. These findings may be associated with the limited number of patients and heterogeneous patients.

Seminal antioxidants protect spermatogenetic activity by neutralizing levels of ROS^22^. Melatonin is an antioxidant targeted at the mitochondria; therefore, it is suggested as a ROS scavenger and could be used to treat infertility^23^. High levels of ROS are a critical parameter affecting sperm DNA integrity^24^.

In total, 39% of men with idiopathic infertility have high sperm DNA damage^25^. Besides increased levels of ROS, infertile men had lower antioxidant capacity than fertile men^26^. Indeed, Iommiello et al. found a significant positive correlation between increased semen ROS levels and SDF^27^. Since the effect of seminal oxidative stress on spermatogenesis is better understood, interest in natural supplements containing high antioxidant capacity will start to increase^28^. Based on these studies, the clinical use of antioxidant support therapies has gained momentum. Most studies on antioxidant support therapies to reduce ROS levels have decreased in DFI^29,30^. Micic et al. investigated the effect of the use of 1.000 g LC, 0.5 g ALC, 0.725 g fumarate, 1 g fructose, 50 mg citric acid, 10 mg zinc, 20 mg coenzyme Q10, 50 μg selenium, 90 mg vitamin C, and 200 μg folic acid. A total of 175 patients with oligoasthenozoospermia were treated with 1.5 μg for 3 months. It has been shown that higher seminal carnitine and α-glucosidase levels lead to improved progressive sperm motility, an increase in sperm viability, and a decrease in DFI^19^. Similar to all these studies, in which antioxidant support therapy has shown the curative effect on ROS and reducing DFI, a significant decrease in DFI was also observed in our study. In our study, the decrease in DFI rates in Group 2, which did not receive antioxidant food support, was not statistically significant. Despite the statistically significant decrease in DFI in Group 1 (p=0.003), the lack of significant improvement in TAC level may be due to the lower mean TAC level at the beginning of treatment in Group 1 patients compared to Group 2. In addition, the antioxidant paradox should come to mind; the subjects may have overdosed on antioxidants. There are hardly any studies evaluating antioxidant overuse and its related side effects. In a review, it was reported that antioxidant therapy prolonged for 6 months could significantly reduce the amount of ROS and impair sperm function by providing a ROS concentration below the amount of ROS required for cell function^31^. Conversely, a meta-analysis showed that 3 months of antioxidant treatment after varicocelectomy was more effective than 6 months of treatment^32^.

In a study of asthenozoospermic infertile men who showed the failure of antioxidant support therapy for 6 months, it was shown that there was no statistical difference in sperm count, motility, morphology, and viable pregnancy rates compared to the placebo group, and DFI rates were similar between the group^6^. Another study showed that empirical antioxidant treatment was not effective on sperm concentration and DFI but had a curative effect on sperm motility and morphology^33^. In our study, although improvement was found in sperm concentration in both groups (with and without antioxidants), which might be related to significantly increased physical activity, no significant change was observed in other sperm parameters, such as motility and progressive motility.

To the best of our knowledge, no clinical study has examined sperm chromatin condensation regarding the percentage of histone-rich spermatozoa after antioxidant food supplementation therapy. Our study found a significant decrease in the percentage of histone-rich sperm in the third month in Group 1 patients who received antioxidant nutritional support (p<0.001). On the contrary, there was no significant change in sperm chromatin structure in Group 2 (p=0.670). Approximately 10–15% of sperm DNA remains packed with histone during the maturation process of human spermatozoa. On the contrary, 85–90% of the DNA is replaced with protamines, which give more stable properties to sperm chromatin. A higher percentage of histones (abnormal protamination) leads to a decrease in DNA stabilization and an increase in DFI. Abnormal protamination has been shown to reduce fertility capacity^34,35^.

This study has some limitations. Antioxidant treatment protocols in idiopathic male infertility should be explicitly personalized according to each patient's specific needs. For example, measuring the ROS level and adjusting the antioxidant titration may affect the treatment outcome. The fact that the ROS level was not evaluated in our study may be a limiting factor. The limited number of patients and non-homogeneous variables may affect the results of sperm motility, morphology, and TAC values. The study population of both groups is overweight, with a BMI between 25 and 30; therefore, obese men react differently to antioxidant food supplements. Many predisposing factors may cause idiopathic infertility, and these would affect the empirical antioxidant treatment outcomes. Finally, although the positive effects of this treatment method on sperm concentration, DFI, and sperm chromatin condensation have been shown, their effects on pregnancy and live birth rates have not been investigated as another limiting factor.

## CONCLUSION

Antioxidant food supplementation significantly decreases DFI and improves sperm chromatin condensation in idiopathic infertile men. Moderate physical activity increases sperm concentration in idiopathic infertile men.
